# Participation of ezrin in bacterial uptake by trophoblast giant cells

**DOI:** 10.1186/1477-7827-7-95

**Published:** 2009-09-09

**Authors:** Kenta Watanabe, Masato Tachibana, Suk Kim, Masahisa Watarai

**Affiliations:** 1 Department of Veterinary Public Health, Faculty of Agriculture, Yamaguchi University, Yamaguchi 753-8515, Japan; 2 Department of Veterinary Public Health, Gyeongsang National University, Gyeongnam 660-701, Korea

## Abstract

**Background:**

Trophoblast giant (TG) cells are involved in systematic removal of bacterial pathogens from the maternal-fetal interface of the placenta. In particular, TG cells have the ability to take up extracellular antigens by active phagocytosis induced by interferon-gamma (IFN-gamma). We previously reported that heat shock cognate protein 70 (Hsc70) present on the surface of TG cells mediated the uptake of Brucella abortus. However, the mechanism of bacterial uptake by TG cells is not completely understood. Here we identified ezrin, a member of ezrin-radixin-moesin (ERM) protein family, as a molecule associated with Hsc70.

**Methods:**

Mouse TG cells were employed in all experiments, and B. abortus was used as the bacterial antigen. Confirmation of the binding capacity of ERM protein was assessed by pull-down assay and ELISA using recombinant Hsc70 and ERM proteins. Ezrin was depleted using siRNA and the depletion examined by immunoblotting or immunofluorescence staining.

**Results:**

The expression level of ezrin was higher in TG cells than in trophoblast stem (TS) cells, and ezrin knockdown TG cells showed a reduction in bacterial uptake ability. Although tyrosine phosphorylation of ezrin was not related to bacterial uptake activity, localization of Hsc70 on the membrane was affected by the depletion of ezrin in TG cells.

**Conclusion:**

Ezrin associates with Hsc70 that locates on the membrane of TG cells and participates in the bacterial uptake by TG cells.

## Background

Trophoblast giant (TG) cells are polyploid cells differentiated from trophoblast stem (TS) cells by many morphological and functional developments; they form the fetal component of placenta [1]. In particular, TG cells play a crucial role in implantation and forming a diffuse network of blood sinuses [2], and promote maternal blood flow to the implantation site in mice [3]. TG cells are essential for establishment of pregnancy. We previously reported that *Brucella abortus*, an intracellular bacteria that causes abortion in pregnant animals, internalized and replicated in TG cells, specifically in pregnant mice [4]. This study suggested that internalization and replication of *Brucella *in TG cells play a key role in causing abortion and that TG cells are closely linked to the avoidance of maternal immune rejection.

Trophoblast cells have a phagocytic ability. During implantation, trophoblast cells invade maternal tissue by phagocytosing the uterine epithelial cells and stroma [5]. Several molecular mechanisms involved in phagocytosis by trophoblast cells have been reported [6], but the complete process remains unclear. It has also been reported that trophoblast cells can phagocytose pathogens and that this activity is enhanced by interferon-gamma (IFN-γ treatment) [7,8]. Therefore, trophoblast cells may act in a manner similar to that of macrophages in phagocytosis. These studies suggested that trophoblast cells play a role in not only development and maintenance of placenta but also in the defense system of placenta.

Heat shock cognate protein 70 (Hsc70) is a member of the 70-kDa heat shock protein (Hsp70) family. The Hsp70 family is comprised of stress-inducible Hsp70 and constitutively expressed Hsc70. Despite the fact that it shares 80-90% sequence identity at the amino acid level with Hsp70, Hsc70 has some functional differences compared to the other members of the Hsp70 family. Hsc70 acts as a multifunctional cytoplasmic chaperone protein to fold and assemble newly synthesized proteins, and sort the proteins to different subcellular compartments [9]. In addition, Hsc70 has some characteristic functions such as preventing apoptosis in the neurulating embryo [10], presentation of antigens with MHC class II [11], and chaperone-mediated autophagy [12].

Hsc70 is located in different subcellular compartments; it is found in the nuclear, cytosol, and cellular membrane. Moreover, cytoplasmic Hsc70 shuttles between the nucleus and the cytoplasm [13,14]. It has also been suggested that Hsc70 is released from the cells subjected to heat stress or other cytotoxic treatments, although Hsc70 has no secretion leader signal [15,16]. Rotavirus reportedly enter host cells using membrane-localized Hsc70 as a receptor [17]. In addition, we have found that Hsc70 is present on the membrane of TG cells, and this is utilized by *B.abortus *to enter TG cells [18]. It has also been reported that Hsc70 can bind fatty acids and bacterial LPS non-covalently [19,20]. These studies support the hypothesis that Hsc70 may participate in the uptake of extracellular antigens by TG cells, and play a principal role in immune function. However, it is unclear how Hsc70 presents on the membrane or acts in TG cells.

Clarification of the function of Hsc70 in TG cells and the phagocytic mechanism of TG cells in pregnant animals requires further investigation. In this study, we identified ezrin, a member of the family of ezrin-radixin-moesin (ERM) proteins, associated with Hsc70 that is located on the TG cell membrane, and investigated the role of ezrin and Hsc70 in bacterial uptake by TG cells.

## Methods

### Cell culture

TS cells were cultured in TS medium in the presence of FGF4, heparin, and mouse embryonic fibroblast (MEFs)-conditioned medium as described previously [18]. The TS medium was prepared by adding 20% fetal bovine serum (FBS), 1 mM sodium pyruvate, 100 μM β-mercaptoethanol, and 2 mM L-glutamine to RPMI 1640. To induce differentiation to TG cells, the cells were cultured in the TS medium alone for 3 days (about 80% of cells were differentiated to TG cells) at 37°C in a CO_2 _incubator. J774 cells were cultured in RPMI 1640 containing 10% fetal FBS.

### Bacterial strains

All *B. abortus *derivatives were from 544 (ATCC23448) smooth virulent *B. abortus *biovar 1 strains. Ba600 (544 GFP+) was used in this study [4]. *B. abortus *strains were maintained as frozen glycerol stocks and cultured on Brucella broth (Becton Dickinson, Franklin Lakes, New Jersey, USA) or Brucella broth containing 1.5% agar.

### Identification of protein that interacts with Hsc70

TG cells (3 × 10^5^/ml) were seeded into each well of a 6-well plate. Protein isolations for the cytoskeleton, nucleus, membrane, and cytosol fractions were performed using the ProteoExtract Subcellular Proteome Extraction kit as described by the manufacturer (Calbiochem, Darmstadt, Germany). The membrane fraction proteins of TG cells (50 μg/ml) were separated on 8% and 12% polyacrylamide gels and transferred to a polyvinylidene fluoride (PVDF) membrane, which was incubated for 1 h at room temperature with peroxidase (POD)-conjugated Hsc70 at a dilution of 1:2000 in phosphate-buffered saline (PBS). POD-conjugated Hsc70 was prepared using the Peroxidase Labeling Kit (Dojindo Molecular Technologies Inc., Kumamoto, Japan). The proteins reacting with Hsc70 were extracted and identified by means of nano LC-MS/MS analysis and MASCOT database searches (APRO life Science Institute, Tokushima, Japan).

### Expression and purification of recombinant proteins

The total RNA of TG cells was isolated using an RNA purification kit (Qiagen, Hilden, Germany). RNA was quantified by absorption at 260 nm using SmartSpec3000 spectrophotometer (Bio-Rad, Hercules, California, USA). RT-PCR was carried out using a Superscript II kit (Invitrogen, Carlsbad, California, USA). The following primers were used for cloning each gene, ezrin, 5'-**GAATTC**ATGCCCAAGCCAATCAACGTCCGG-3' (*Eco*RI site underlined) and 5'-**GTCGAC**CTACATGGCCTCGAACTCGTCAAT-3' (*Sal*I site underlined); radixin, 5'-**CTCGAG**ATGCCGAAGCCAATCAATGTAAGA-3' (*Xho*I site underlined) and 5'-**GTCGAC**TCACATGGCTTCAAACTCATCGAT-3' (*Sal*I site underlined); moesin, 5'-**CTCGAG**ATGCCGAAGACGATCAGTGTGCGT-3' (*Xho*I site underlined) and 5'-**GTCGAC**CTACATGGACTCAAACTCATCAAT-3' (*Sal*I site underlined); Hsc70 full length, 5'-**CTCGAG**ATGTCTAAGGGACCTGCAGTT-3' (*Xho*I site underlined) and 5'-**GTCGAC**TTAATCCACCTCTTCAATGGT-3' (*Sal*I site underlined); Hsc70 ATP binding domain, 5'-**CTCGAG**ATGTCTAAGGGACCTGCAGTT-3' (*Xho*I site underlined) and 5'-**GTCGAC**AGACTTGTCTCCAGATAGAAT-3' (*Sal*I site underlined); and Hsc70 peptide-binding domain, 5'-**CTCGAG**GAGAACGTTCAGGATTTGCTG-3' (*Xho*I site underlined) and 5'-**GTCGAC**TTAATCCACCTCTTCAATGGT-3' (*Sal*I site underlined). These products were cloned into the pCold TF vector (Takara Bio Inc., Shiga, Japan). The His-tagged recombinant proteins were expressed in the *Escherichia coli *strain DH5α, and purified by Ni-NTA chromatography (Qiagen). The His-tagged proteins were cleaved using human rhinovirus 3C protease according to the manufacturer's instructions (Novagen, Darmstadt, Germany).

### Immunoblotting

The cell lysates were separated on 10% polyacrylamide gels and transferred to a PVDF membrane, which was incubated for 1 h at room temperature with primary antibody, anti-Hsc70 rat monoclonal antibody (mAb) (SPA-815; Stressgen, Victoria, BC, Canada) at a dilution of 1:5000 and anti-ezrin, anti-radixin, and anti-moesin rabbit mAb (EP924Y, EP1862Y, and EP1863Y; EPITOMICS, Burlingame, California, USA) at a dilution of 1:2000 in 5% skim milk. It was then washed three times in Tris-buffered saline (TBS) with 0.02% Tween 20, incubated for 30 min with an HRP-conjugated secondary antibody at 0.01 μg/ml, and then washed again. Immunoreactions were visualized using the enhanced chemiluminescence detection system (GE Healthcare Life Science, Little Chalfont, UK).

### Pull-down assay

For the pull-down assay, protein G agarose bead-bound mAbs for each protein (20 μg/ml) were added to 1 ml of purified Hsc70 or each ERM protein mixture (20 μg/ml) in PBS and the mixture was incubated at 37°C for 2 h. The precipitates were washed with PBS, analyzed by means of sodium dodecyl sulfate-polyacrylamide gel electrophoresis (SDS-PAGE), and stained with Coomassie Brilliant Blue (Wako, Osaka, Japan).

### ELISA

The ability of ERM proteins to bind Hsc70 was measured as follows. A 100-μl aliquot of each ERM protein (10 μg/ml) was placed in the wells of 96-well immunoplates (Nalge Nunc, Rochester, NY, USA) and incubated at room temperature for 2 h. The samples were then removed, and the wells were washed twice with PBS-0.05% Tween 20. PBS containing 5% bovine serum albumin (BSA) was added in each well for blocking and incubated at 37°C for 30 min. A 100-μl aliquot of Hsc70 (20 μg/ml) was added and the plate was incubated at 37°C for 2 h. The amount of bound Hsc70 was determined using ELISA with anti-Hsc70 rat mAB.

### Immunofluorescence microscopy

TG cells grown on coverslips in 12-well plates were washed twice with PBS, fixed in 4% paraformaldehyde for 30 min at room temperature and permeabilized in 0.2% or 0.01% Triton X-100. After washing three times with PBS, samples were incubated with primary antibodies: anti-Hsc70 (Stressgen), anti-ezrin (EPITOMICS), and anti-p-Ezrin (Tyr 146 and 354) (Santa Cruz Biotechnology, Inc., Santa Cruz, CA, USA) antibodies at 1:200 dilution, or Alexa Fluor 594-phalloidin (Molecular Probes, Eugene, Oregon, USA) at 20 μg/ml in PBS for 60 min at 37°C. After washing three times with PBS, samples were incubated with TRITC or FITC-labeled goat anti-rat or anti-rabbit IgG at 0.01 μg/ml in PBS at 37°C for 60 min. Samples were then placed in mounting medium (90% glycerol containing 1 mg/ml phenylenediamene in PBS, pH 9.0) and visualized by fluorescence microscopy.

### Small interfering RNA experiment

The small interfering RNA (siRNA) duplexes used for silencing mouse ezrin (target sequence: TTCGGAGATTATAACAAGGAA), radixin (target sequence: TGGCTAGGTGTTGATGCTTTA), and AllStars Negative Control siRNA were purchased from QIAGEN. TG cells were transiently transfected using oligofectamine (Invitrogen) with or without siRNA at final concentration of 10 nM. The treatment with the siRNA and oligofectamine had little effect on cell viability or ability of adherence in TG cells.

### Efficiency of bacterial uptake by TG cells

Uptake activity of bacteria by TG cells was measured using a modified version of the method of Watanabe *et al *[18]. TG cells grown in 24-well plates were treated with recombinant interferon-gamma (IFN-γ) (Cedarlane Laboratories, Ontario, Canada) at 1000 U/ml for 12 h before inoculation of bacteria to promote the uptake for heat-killed bacteria. Alive or heat-killed *B. abortus *(1 × 10^6^) were deposited onto the TG cells by centrifugation at 150 × g for 10 min at room temperature, and the cells were incubated for 30 min at 37°C in a CO_2 _incubator. To measure uptake efficiency for live *B. abortus*, the cells were washed once with PBS and then were incubated with RPMI 1640 with gentamicin (30 μg/ml) for 30 min to kill the extracellular bacteria. The cells were then washed three times with PBS and then were lysed with distilled water. Colony forming units (CFU) were measured by serial dilutions on Brucella agar plates. For killed *B. abortus*, after 30 min of incubation, the cells were washed three times with PBS and fixed in 4% paraformaldehyde for 30 min at room temperature. The number of bacteria within cells was counted using microscope. To prepare cholesterol-depleted cells, TG cells were treated with methyl-β-cyclodextrin (MβCD) (Wako) at the indicated concentrations for 30 min.

### Statistical analyses

All statistical analyses were conducted using Student's *t*-test.

## Results

### Ezrin interacts with Hsc70 on TG cell membrane

**Figure 1:**
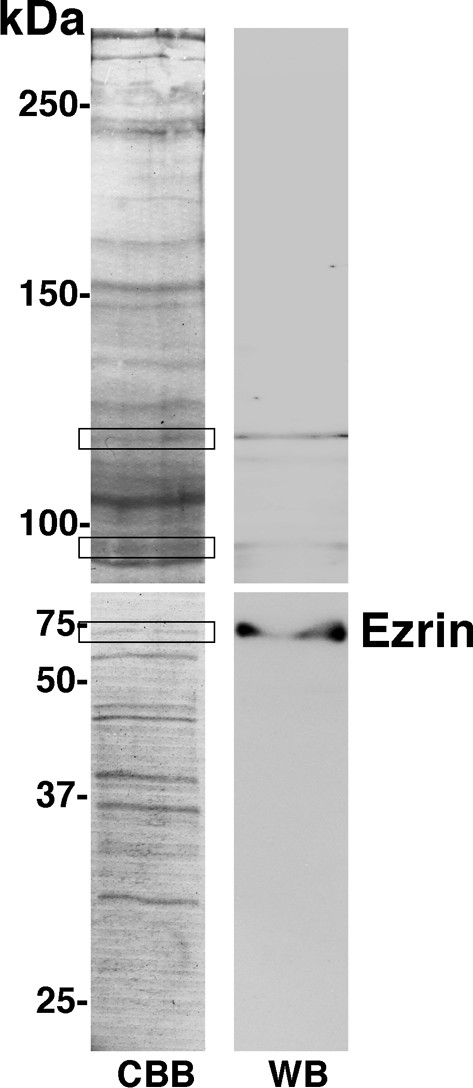
**Identification of the molecule that interacts with Hsc70**. TG cell membrane fraction proteins were separated by SDS-PAGE and stained with Coomassie brilliant blue (CBB), or immunoblotted with POD-conjugated Hsc70 (WB). The three bands that showed reactivity in WB are surrounded with frames.

We previously reported that Hsc70 localized on TG cell membranes and contributed to bacterial uptake [18]. To clarify the functional significance of this inference, we first tried to identify the molecules that interact with Hsc70 in TG cell membranes by mass spectrometry analysis. The membrane fraction proteins of TG cells were separated by SDS-PAGE and transferred to a PVDF membrane. The protein reacting most strongly with POD-conjugated Hsc70 around the 75-kDa single band was extracted from the PVDF membrane and subjected to LC-MS/MS analysis. A MASCOT database search identified it as ezrin (Fig. 1). It was impossible to identify the other two bands because of contamination of many kinds of proteins by same method.

### Ezrin is able to bind to Hsc70 more strongly than other ERM family proteins

**Figure 2:**
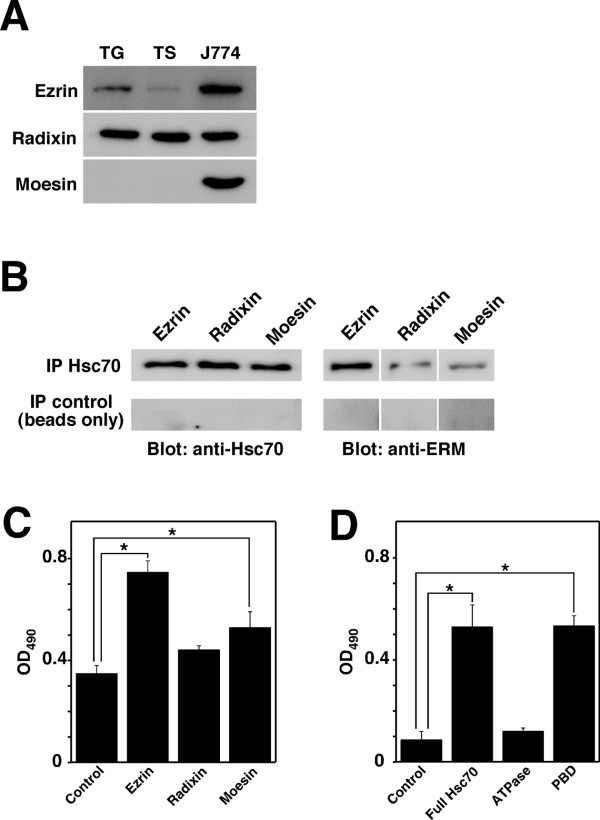
**Expression of ERM proteins in TG cells, and the binding capacity between ezrin and Hsc70**. (A) Expression of ERM proteins in TG, TS, and J774 macrophage cells. Immunoblot analyses were performed with anti-ezrin, anti-radixin, and anti-moesin antibodies. (B) Affinity of ERM proteins for Hsc70 shown by pull-down assay. Recombinant Hsc70 and each ERM protein were mixed, and these samples were immunoprecipitated with anti-Hsc70 antibodies or beads only. Detection of proteins was performed by immunoblotting. (C) The binding capacity of ERM proteins to Hsc70 were measured by ELISA. Immunoplates were coated with each ERM protein or BSA (control), and then Hsc70 was added. Data are the averages of triplicate samples from three identical experiments. Error bars represent standard deviation. Statistically significant differences between control and ERM proteins are indicated by asterisks (*, P < 0.01). (D) ELISA was used to determine the binding domain of Hsc70 for ezrin. Immunoplates were coated with full-length Hsc70 (Full Hsc70), ATP-binding domain (ATPase), and peptide-binding domain (PBD) of Hsc70, following which ezrin was added. Data are the averages of triplicate samples from three identical experiments. Error bars represent standard deviation. Statistically significant differences between control and each domain proteins are indicated by asterisks (*, P < 0.01).

**Figure 3:**
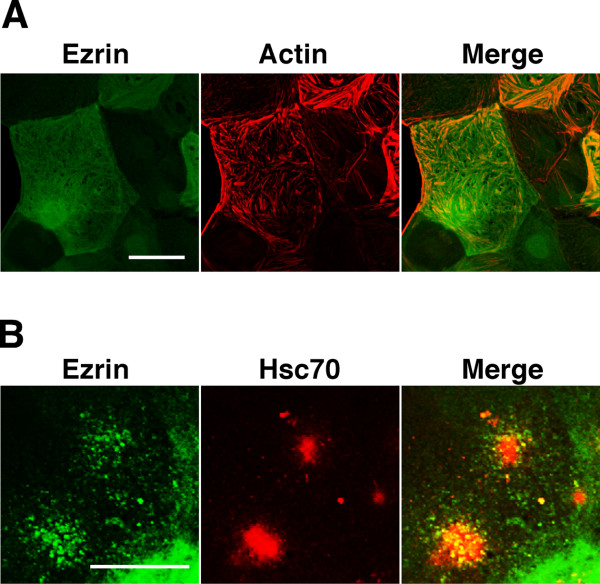
**Distribution of ezrin in TG cells**. Immunofluorescence staining of ezrin (green) and F-actin (red) in TG cells permeabilized with 0.2% Triton-X100 (A), and Hsc70 (red) in TG cells moderately permeabilized with 0.01% Triton-X100 (B). Scale bar indicates 50 μm.

The ERM family consists of three closely related proteins; sequencing of cDNAs has revealed that the amino acid sequence identity among the ERM proteins is 70-80% [21]. We therefore investigated the expression of ERM proteins in TG cells, TS cells, and J774 macrophage cells by immunoblotting. All ERM proteins were detectable in J774 cells, but only moesin was not detected in TS and TG cells (Fig. 2A). The expression level of ezrin in TG cells was higher than that in TS cells. Next, to confirm the ability of ERM proteins to bind to Hsc70, we tested them by pull-down assay with each recombinant ERM proteins or Hsc70. Immunoblotting of the precipitated proteins showed the association between ERM proteins and Hsc70, but there was no association when only beads were present (Fig. 2B). The association was also confirmed by ELISA using ERM protein-coated immunoplates. Hsc70 bounded to the ezrin-coated wells most strongly (Fig. 2C). It has been reported that Hsc70 contains three functional domains, i.e., ATP-binding domain (ATPase), the peptide-binding domain (PBD), and the carboxy-terminal domain (CTD) [22]. To identify the binding domain of Hsc70, the full-length, ATPase domain, and peptide-binding domain of Hsc70 were constructed, and the ability of each recombinant Hsc70 to bind to ezrin was confirmed by ELISA. Ezrin reacted with PBD of Hsc70 (Fig. 2D). Furthermore, we examined the localization of ezrin in TG cells. Using Triton X-100, it was observed that ezrin co-localized with the actin cytoskeleton inside permeabilized TG cells, and with Hsc70 on TG cell membranes (Fig. 3).

### Ezrin contributes to bacterial uptake by TG cells

**Figure 4:**
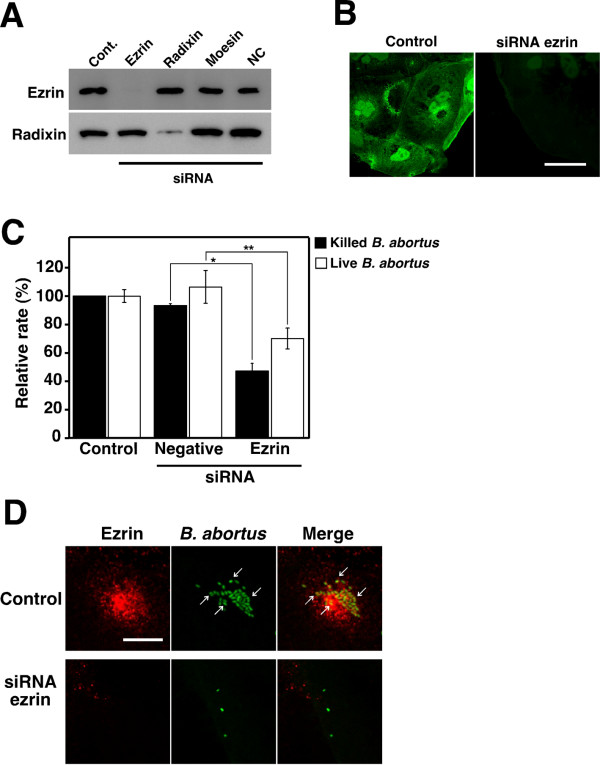
**Effect of depletion of ezrin on bacterial uptake by TG cells**. (A) TG cells were treated for 48h with siRNA targeting ezrin or without it (Cont.), radixin, moesin, or the negative control siRNA (QIAGEN AllStars Negative Control) (NC). Depletion of ezrin was examined by immunoblotting. Radixin was used as a comparable control. (B) Distribution of ezrin in non-treated (Control) and ezrin-depleted (siRNA ezrin) TG cells. Scale bar indicates 50 μm. (C) Bacterial uptake by TG cells. Killed or live *B. abortus *were deposited onto ezrin-depleted TG cells. TG cells were also treated with IFN-γ at 1000 U/ml for 12 h before inoculation of heat-killed bacteria. This result of ezrin depletion corresponds to panels A and B. Data are the averages of triplicate samples from three identical experiments. Error bars represent standard deviation. Statistically significant differences from control are indicated by asterisks (*, P < 0.01 and **, P < 0.05). (D) Distribution of ezrin (red) and heat-killed *B. abortus *(green) in non-treated (Control) and ezrin-depleted (siRNA ezrin) TG cells after 30 min of bacterial inoculation. TG cells were also treated with IFN-γ at 1000 U/ml for 12 h before inoculation of bacteria. Arrows point to co-localized bacteria. Scale bar indicates 10 μm.

To examine the effect of ezrin on bacterial uptake by TG cells, we used siRNA to knock down ezrin in the TG cells. The expression level of ezrin was no longer detectable, but was not affected by transfection with radixin or control siRNA (Fig. 4A and 4B). Using heat-killed and live *B. abortus *as bacterial antigens, the uptake efficiency by TG cells was significantly reduced by transfection with ezrin-specific siRNA (Fig. 4C and 4D). There is no significant change of bacterial uptake activity by knockdown of radixin (data not shown).

### Bacterial uptake was independent of ezrin phosphorylation

**Figure 5:**
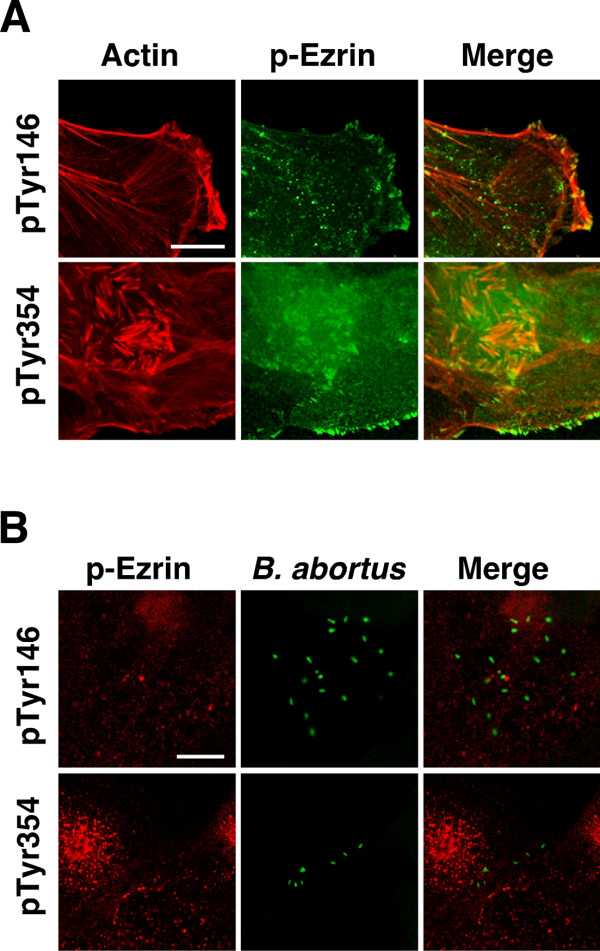
**Distribution of tyrosine-phosphorylated ezrin in TG cells**. (A) Immunofluorescence staining of F-actin and tyrosine-phosphorylated ezrin (p-Ezrin). Anti-phosphorylated tyrosine residues 146 (pTyr146) and 354 (pTyr354) of ezrin were used to detect p-Ezrin. Scale bar indicates 25 μm. (B) Immunofluorescence staining of p-Ezrin (red) and *B. abortus *(green). Cells were observed after 30 min of bacterial inoculation. Scale bar indicates 10 μm.

To evaluate whether ezrin phosphorylation is a requirement for bacterial uptake, we tried to detect tyrosine phosphorylation of ezrin at the site of bacterial uptake in TG cells. Although tyrosine-phosphorylated ezrin was strongly co-localized with the actin cytoskeleton (Fig. 5A), co-localization between phosphorylated ezrin and intracellular bacteria was not observed (Fig. 5B). The change in quantity of tyrosine phosphorylation of ezrin due to bacterial inoculation was not detected by immunoblotting (data not shown).

### Ezrin supports Hsc70 localization on TG cell membrane

**Figure 6:**
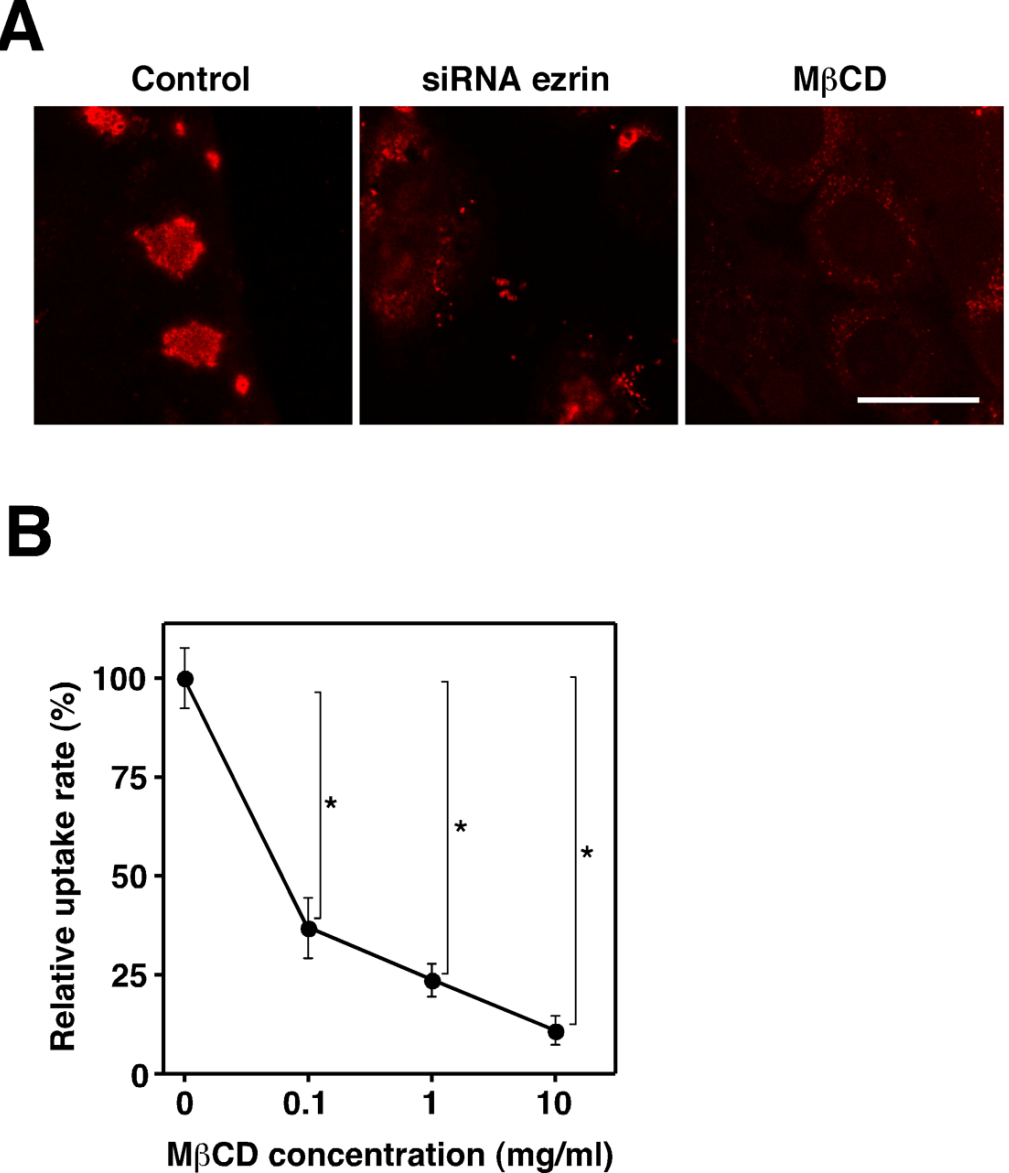
**Effect of MβCD treatment and depletion of ezrin on distribution of Hsc70**. (A) Immunofluorescence staining of Hsc70 on TG cells that were not treated (Control), TG cells with reduced endogenous ezrin by siRNA (siRNA ezrin), and TG cells treated with 10 mg/ml MβCD (MβCD). Scale bar indicate 50 μm. (B) Heat-killed *B. abortus *were deposited onto TG cells that were treated with MβCD at the indicated concentrations for 30 min. The uptake efficiency is shown as relative uptake rate. Data are the averages of triplicate samples from three identical experiments. Error bars represent standard deviation. Statistically significant differences from TG cells that were not treated are indicated by asterisks (*, P < 0.01).

It was reported that Hsc70 localized on the membrane by rafting on the cholesterol rich microdomains. Methyl-β-cyclodextrin (MβCD) treatment has been shown to affect the distribution of Hsc70 on the membrane [23]. We investigated the effect of MβCD on Hsc70 distribution and bacterial uptake in TG cells. MβCD treatment significantly decreased Hsc70 distribution on the membrane (Fig. 6A). In addition, bacterial uptake was found to be inhibited dependently on MβCD concentration (Fig. 6B). Ezrin-Hsc70 co-localization was not detected on the membrane of TG cells that were treated with MβCD (data not shown). Moreover, the distribution of Hsc70 on the membrane was affected by ezrin knockdown in TG cells (Fig. 6A).

## Discussion

Members of the ERM protein family are known as membrane-associated proteins responsible for linking the membrane to the actin cytoskeleton [24]. Membrane proteins such as CD43, CD44, and intercellular adhesion proteins (ICAMs) are reported to interact with ezrin [25-27]. In this study, we demonstrated that ezrin interacts with Hsc70 localized on membrane of TG cells, and contributes to bacterial uptake by TG cells.

Ezrin is highly expressed in epithelial cells. Many studies have reported that ezrin plays roles in morphological changes by arranging the cytoskeleton organization [28-30]. In TG cells, the expression level of the endogenous ezrin protein was higher than in TS cells. This result supports the findings of a previous study that actin stress fibers also develop by differentiation of TG cells [1]. Ezrin is also thought to be an essential molecule for internalization of several intracellular pathogens that are characterized by the rearrangement of actin cytoskeleton, and can be detected at the invasion sites of these pathogens, *Shigella*, *Listeria*, and *Neisseria *[31-33]. In this study, we demonstrated that internalization efficiency of *Brucella *was reduced significantly by ezrin knockdown in TG cells. This result suggested that internalization of *Brucella *is also mediated by endogenous ezrin. However, the relative decrease in the rate of internalization of live *Brucella *due to depletion of ezrin was lower than that of killed *Brucella *in TG cells. It suggested that *Brucella *invades the TG cells in several ways in addition to the ezrin-related route. We previously reported that the dramatic actin polymerization at the invasion sites of *Brucella *was not observed in TG cells [18]. Therefore, the activation of ezrin induced by internalization of *Brucella *may not occur, or be separate from the induction of actin polymerization event in TG cells.

Ezrin is thought to be a substrates for some kinds of tyrosine kinase [34], and is phosphorylated transiently on tyrosine residues 146 and 354; it is also involved in the antigen-receptor-mediated tyrosine phosphorylation pathway in lymphocytes [35]. This tyrosine phosphorylation plays a role in cell activation and transformation [36]. Moreover, it is reported that the tyrosine phosphorylation of ezrin occurs in response to bacterial invasion. However, not all intracellular pathogens trigger tyrosine phosphorylation of ezrin by their internalization [37,38]. We could not detect upregulation of tyrosine-phosphorylated ezrin during bacterial uptake in TG cells (data not shown), and there was no tyrosine phosphorylation of ezrin at the site of bacterial uptake. Thus, interaction between Hsc70 and ezrin as well as the bacterial uptake may be independent of the tyrosine phosphorylation of ezrin.

Hsc70 has been reported to be present on the surface of several types of cells [20]. However, it is not clear how Hsc70, which does not have a membrane-spanning domain, presents on the membrane. Several molecules have been suggested as receptors for the Hsp70 family, such as CD14, CD40, CD91, and the scavenger receptor Lox-1 [39-41]. Hsc70 may present on the cell surface by associating with such receptors in TG cells. Meanwhile, it has been reported that Hsc70 localized on membranes by direct association with lipid rafts or cholesterol rich microdomains [42,43]. We observed that Hsc70 on TG cells membranes were affected by MβCD treatment, and knockdown of ezrin by siRNA also prevented the distribution of Hsc70 on the membrane. According to these results, Hsc70 may be able to present on membranes or lipid raft microdomains by interacting with ezrin. By forming a functional complex with ezrin, Hsc70 may allow the uptake of antigens by TG cells. Thus, depletion of ezrin caused a deficiency in the function of Hsc70 on TG cell membranes, and this is likely the reason why ezrin knockdown impaired bacterial uptake by TG cells. However, since ezrin can interact with many other molecules in the membrane or cytoplasm, as described above, the possibility that the interaction between ezrin and some other molecule may affect the internalization efficiency of *Brucella *into TG cell remains an issue to be considered.

## Conclusion

The findings of this study that ezrin contributes to bacterial uptake by TG cells provides new information on TG cells, which can be applied in the understanding of the immune mechanism involved in pregnancy. However, it is not known whether the results observed in this study are generally applicable. Further studies on the participation of ezrin in TG cell function will reveal the mechanism of TG cells involved in immune systems in pregnancy and help the development of methods contributing to the success of pregnancies.

## Competing interests

The authors declare that they have no competing interests.

## Authors' contributions

MW conceived the study. MW and KW designed the experiments, interpreted the results, and worked on the manuscript. KW and MT carried out most of the experimental works. KS participated in cell culture and experiments of infection. All authors read and approved the final manuscript.
